# Proteasome-associated HECT-type ubiquitin ligase activity is required for plant immunity

**DOI:** 10.1371/journal.ppat.1007447

**Published:** 2018-11-20

**Authors:** James J. Furniss, Heather Grey, Zhishuo Wang, Mika Nomoto, Lorna Jackson, Yasuomi Tada, Steven H. Spoel

**Affiliations:** 1 School of Biological Sciences, University of Edinburgh, Edinburgh, United Kingdom; 2 The Center for Gene Research, Division of Biological Science, Nagoya University, Nagoya, Japan; Texas A & M University, UNITED STATES

## Abstract

Regulated degradation of proteins by the 26S proteasome plays important roles in maintenance and signalling in eukaryotic cells. Proteins are marked for degradation by the action of E3 ligases that site-specifically modify their substrates by adding chains of ubiquitin. Innate immune signalling in plants is deeply reliant on the ubiquitin-26S proteasome system. While progress has been made in understanding substrate ubiquitination during plant immunity, how these substrates are processed upon arrival at the proteasome remains unclear. Here we show that specific members of the HECT domain-containing family of ubiquitin protein ligases (UPL) play important roles in proteasomal substrate processing during plant immunity. Mutations in *UPL1*, *UPL3* and *UPL5* significantly diminished immune responses activated by the immune hormone salicylic acid (SA). In depth analyses of *upl3* mutants indicated that these plants were impaired in reprogramming of nearly the entire SA-induced transcriptome and failed to establish immunity against a hemi-biotrophic pathogen. UPL3 was found to physically interact with the regulatory particle of the proteasome and with other ubiquitin-26S proteasome pathway components. In agreement, we demonstrate that UPL3 enabled proteasomes to form polyubiquitin chains, thereby regulating total cellular polyubiquitination levels. Taken together, our findings suggest that proteasome-associated ubiquitin ligase activity of UPL3 promotes proteasomal processivity and is indispensable for development of plant immunity.

## Author summary

Plants are continuously exposed to different disease agents, including bacteria, fungi, oomycetes and chewing or sucking insects. To protect themselves plants have evolved a sophisticated multi-layered immune system that depends on the reprogramming of large gene repertoires to prioritize the expression of immune genes over normal cellular household genes. Activity of the proteasome, a large proteolytic complex that degrades proteins, is vital to coordinate the expression of immune genes. While it is well understood that proteins marked by a chain of the small polypeptide ubiquitin can be targeted to the proteasome for degradation, it remains unclear how these proteins are processed by proteasomes. Here we identify the enzyme UPL3 that enabled plant proteasomes themselves to add further ubiquitin chains to cellular proteins destined for degradation. This is thought to be an important activity that increases the affinity of substrates for proteasomes while preventing them from stalling during degradation. Importantly, we show that this activity of UPL3 is indispensable for gene expression reprogramming and establishment of disease resistance. Thus, by enabling proteasomes to add ubiquitin marks to its substrates, UPL3 regulates key aspects of plant immunity that could be further exploited in future crop protection strategies.

## Introduction

The ubiquitin-26S proteasome system (UPS) plays an essential cellular role in selective degradation of proteins that are short-lived or damaged. Degradation of proteins is mediated by an enzymatic cascade in which a small and highly conserved ubiquitin molecule is covalently attached to the substrate. Typically an ubiquitin-activating E1 enzyme forms a high-energy thioester bond to an ubiquitin adduct, which is then transferred onto the active site of an ubiquitin conjugating E2 enzyme. In partnership with an E3 ligase that recruits a specific substrate, the E2 enzyme facilitates formation of an isopeptide bond between the ε-amino group of a lysine residue within the substrate and the carboxy-terminal group of ubiquitin. Reiterations of this reaction cycle result in subsequent ubiquitin molecules being similarly attached to internal lysines of the preceding ubiquitin moiety, thereby generating a polyubiquitin chain on the substrate [1, 2]. Lysine 48-linked chains of four or more ubiquitins show high affinity for ubiquitin receptors within the 19S regulatory cap of the proteasome [3]. Substrate degradation involves its unfolding by chaperone activity of the 19S particle, cleavage and release of the polyubiquitin chain for recycling, and subsequent threading of the unfolded substrate into the 20S subunit of the proteasome, a barrel-shaped multi-catalytic proteinase [4].

In comparison to other eukaryotes, plant genomes often encode for a disproportionally large number of genes related to the ubiquitin-26S proteasome system. Particularly E3 ligases are overrepresented, with the Arabidopsis genome, for example, encoding for over 1,400 different predicted E3 ligase components [5]. Accordingly, protein ubiquitination plays vital roles in numerous aspects of plant biology. Indeed, genetic analyses have shown that many developmental and environmental response pathways exhibit a high degree of dependency on components of the ubiquitin-mediated proteasomal degradation pathway [5–7]. Over the last decade it has become increasingly clear that plant immune responses are particularly dependent on ubiquitin-mediated protein degradation [8–11]. Basal resistance as well as race-specific pathogen resistance triggered by intracellular NLR (nucleotide-binding/leucine-rich repeat) immune receptors was compromised by mutation of UBA1, one of two ubiquitin-activating E1 enzymes in Arabidopsis [12]. Similarly, a screen for ubiquitin conjugating E2 enzymes in tomato revealed important roles for a subset of these enzymes in both local immunity and pathogen effector-induced suppression of immune responses [13]. Furthermore, various E3 ligases of the RING and Plant U-box (PUB) types have been identified to play both positive and negative roles in orchestration of plant immune responses [8–11]. Whereas several PUB ligases regulate signalling by pathogen pattern recognition receptors, RING-type E3 ligases have been shown to regulate the proteins levels of NLR immune receptors. Levels of the NLR receptors SNC1 and RPS2 are regulated by the RING-type modular SCF^CPR1^ (*i*.*e*. SKP1/Cullin1/F-box) E3 ligase in which the F-box protein, CPR1 (*c*onstitutive expressor of *p*athogenesis-*r*elated (*PR*) genes 1), functions as the substrate adaptor that recruits these NLR receptors [14, 15]. Failure to degrade these and other NLR receptors can lead to their excessive accumulation, which is associated with spontaneous cell death in absence of pathogen threat [16–21], emphasising the importance of E3 ligases in cellular decisions of life and death.

Ubiquitination also plays key roles in signalling by the immune hormone salicylic acid (SA). Upon pathogen recognition SA accumulates in both local and systemic tissues where it induces profound changes in gene expression to prioritise immune responses over other cellular functions [22]. SA-induced transcriptional reprogramming is mediated by the transcription coactivator NPR1 (*n*onexpressor of *PR* genes), a master regulator of plant immunity [23]. Mutation of *NPR1* renders plants completely insensitive to SA and consequently defective in local and systemic immune responses [24–27]. Interestingly, transcription coactivator activity of NPR1 is regulated by its signal-induced degradation in the nucleus. In absence of pathogen threat, NPR1 activity is continuously restricted by proteasome-mediated clearance from the nucleus, thereby preventing untimely immune gene expression [28]. Instead of stabilising NPR1, unexpectedly SA was found to facilitate recruitment of NPR1 to a modular multi-subunit Cullin-RING-Ligase 3 (CRL3) [28, 29]. Importantly, CRL3-mediated ubiquitination and turnover of NPR1 was necessary for the SA-induced transcriptional activation of its target genes. Taken together, these findings underline the importance of the ubiquitin-26S proteasome system in regulating diverse aspects of plant immune signalling.

Upon arrival at the proteasome, ubiquitinated substrates may be extensively remodelled by various proteasome-associated ubiquitin chain modifying enzymes, including ubiquitin ligases of the HECT-type family [30, 31]. This family of ligases utilise a conserved cysteine residue in the HECT domain that forms a covalent thioester bond with ubiquitin before it is transferred onto the substrate. The ubiquitin remodelling activities of some HECT-type ligases are thought to increase proteasome processivity [32–35]. Given the indispensable roles protein ubiquitination plays in plant immunity, we investigated if HECT-type ubiquitin ligases are involved in proteasome-mediated degradation during immune signalling. Here we report that specific HECT-type ubiquitin ligases of the Ubiquitin Protein Ligase (UPL) family regulate SA-mediated plant immune signalling. In particular we show that UPL3 associated with proteasomal degradation pathway components and provided the proteasome with ubiquitin ligase activity, which was necessary for large scale SA-induced transcriptional reprogramming and immunity. These data suggest that UPL3 plays a vital role in promoting immune-related proteasomal processivity.

## Results

### UPL6 and UPL7 are not involved in SA-dependent plant immune responses

**Fig 1:**
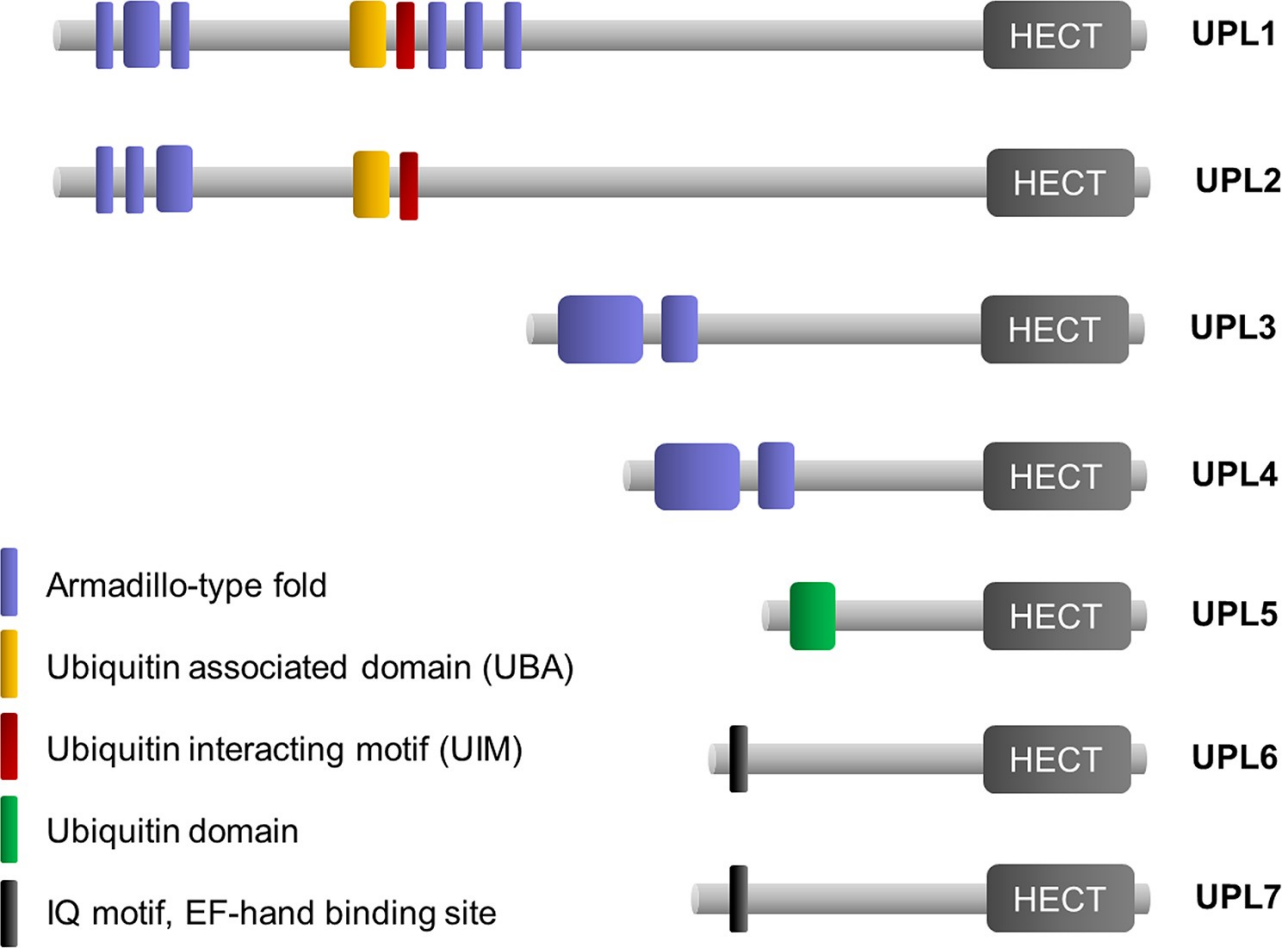
Protein domains in the family of UPL ubiquitin ligases. The family of UPL ubiquitin ligases consists of seven members with diverse N-terminal domains. The C-terminal end of all UPLs contains the HECT domain responsible for substrate ubiquitination.

**Fig 2:**
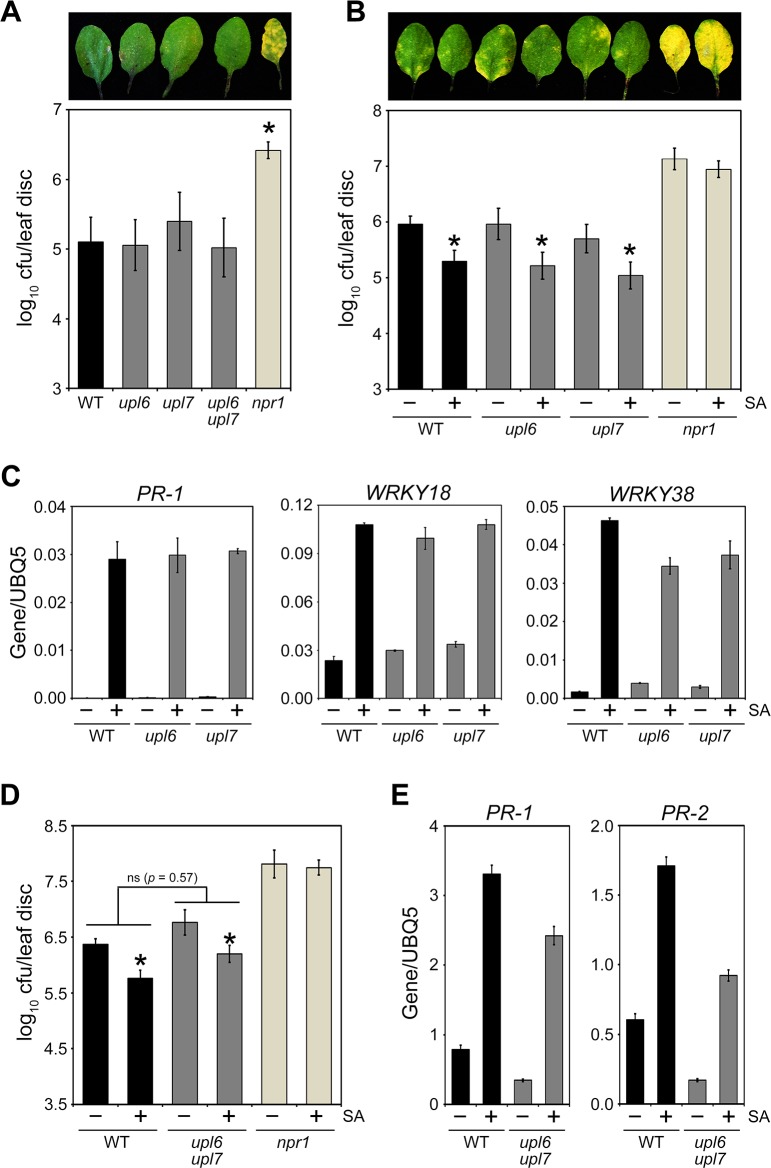
UPL6 and UPL7 are not required for SA-mediated plant immune responses. **(A)** Wild type (WT), *upl6* or *upl7* single and double mutants, and *npr1* plants were infected with *Psm* ES4326 (5 x 10^5^ cells) and disease symptoms (top panel) as well as pathogen growth (bottom panel) assessed after 4 days. Cfu, colony forming units. Error bars represent statistical 95% confidence limits (n = 8) and asterisks indicate statistically significant differences compared to WT (Tukey-Kramer ANOVA test; α = 0.05, n = 8). **(B)** Wild-type (WT), *upl6*, *upl7* and *npr1* plants were treated with 0.5 mM SA for 24 hours after which plants were infected with *Psm* ES4326 (5 x 10^6^ cells) and disease symptoms (top panel) as well as pathogen growth (bottom panel) assessed after 4 days. Cfu, colony forming units. Error bars represent statistical 95% confidence limits (n = 8) and asterisks indicate statistically significant differences between mock (-) and SA (+) treatments for each genotype (Tukey-Kramer ANOVA test; α = 0.05, n = 8). **(C)** Fourteen-day old wild-type (WT), *upl6*, *upl7* and *npr1* seedlings were treated with (+) or without (-) 0.5 mM SA for 6 hours. Expression of the immune marker genes *PR-1*, *WRKY18* and *WRKY38* was analysed by qPCR and normalised against constitutively expressed *UBQ5*. Error bars represent SD (n = 3). **(D)** Wild-type (WT), *upl6 upl7* and *npr1* plants were treated with 0.5 mM SA for 24 hours after which plants were infected with *Psm* ES4326 (5 x 10^6^ cells) and pathogen growth assessed after 4 days. Cfu, colony forming units. Error bars represent statistical 95% confidence limits (n = 8) and asterisks indicate statistically significant differences between mock (-) and SA (+) treatments for each genotype (Tukey-Kramer ANOVA test; α = 0.05, n = 8). No significant difference (ns) was detected between the levels of SA-induced resistance in *upl6 upl7* double mutants compared to WT. **(E)** Adult wild-type (WT) and *upl6 upl7* plants were treated with (+) or without (-) 0.5 mM SA for 24 hours. Expression of the immune marker genes *PR-1* and *PR-2* was analysed by qPCR and normalised against constitutively expressed *UBQ5*. Error bars represent SD (n = 3).

The Arabidopsis UPL family consists of 7 members that all contain a C-terminal HECT domain that accepts ubiquitin from an E2 conjugating enzyme and then transfers it to the target substrate. N-terminal to the HECT domain, UPLs contain different interaction motifs, including ubiquitin-associated (UBA), ubiquitin-like (UBL) and ubiquitin-interacting motifs (UIM), armadillo repeats (ARM), and IQ calmodulin and C-type lectin binding motifs (Fig 1) [36]. As calcium and calmodulin have been implicated in plant defence[37], we first explored if IQ calmodulin binding motif-containing UPL6 and UPL7 proteins play a role in plant immune responses. We generated *upl6* and *upl7* knock-out mutants (S1 Fig) and infected these plants with a low dosage of the bacterial leaf pathogen *Pseudomonas syringae* pv. *maculicola* (*Psm*) ES4326. At this dosage wild-type plants were resistant to this pathogen, while the SA-insensitive *npr1* mutant displayed enhanced disease susceptibility (Fig 2A). Mutant *upl6* and *upl7* plants exhibited similar levels of resistance to *Psm* ES4326 as the wild type. Moreover, *upl6 upl7* double mutants also effectively suppressed the growth of this pathogen, indicating that UPL6 and UPL7 do not regulate basal resistance responses.

To assess if UPL6 and UPL7 regulate induced resistance responses, plants were treated with SA prior to infection with *Psm* ES4326. Whereas SA induced resistance in wild-type plants, it failed to activate defences in mutant *npr1* plants which remained susceptible (Fig 2B). Both *upl6* and *upl7* single mutants as well as *upl6 upl7* double mutant plants displayed normal SA-induced resistance to *Psm* ES4326 (Fig 2B and 2D). This was accompanied by normal levels of SA-induced expression of immune marker genes in single mutants (Fig 2C), while the *upl6 upl7* double mutant was moderately compromised in expression of SA-responsive *PR* genes (Fig 2E). These data suggest that UPL6 and UPL7 ubiquitin ligases play only minor roles in SA-mediated immune responses.

### UPL1 and UPL5 regulate SA-induced gene expression and immunity

**Fig 3:**
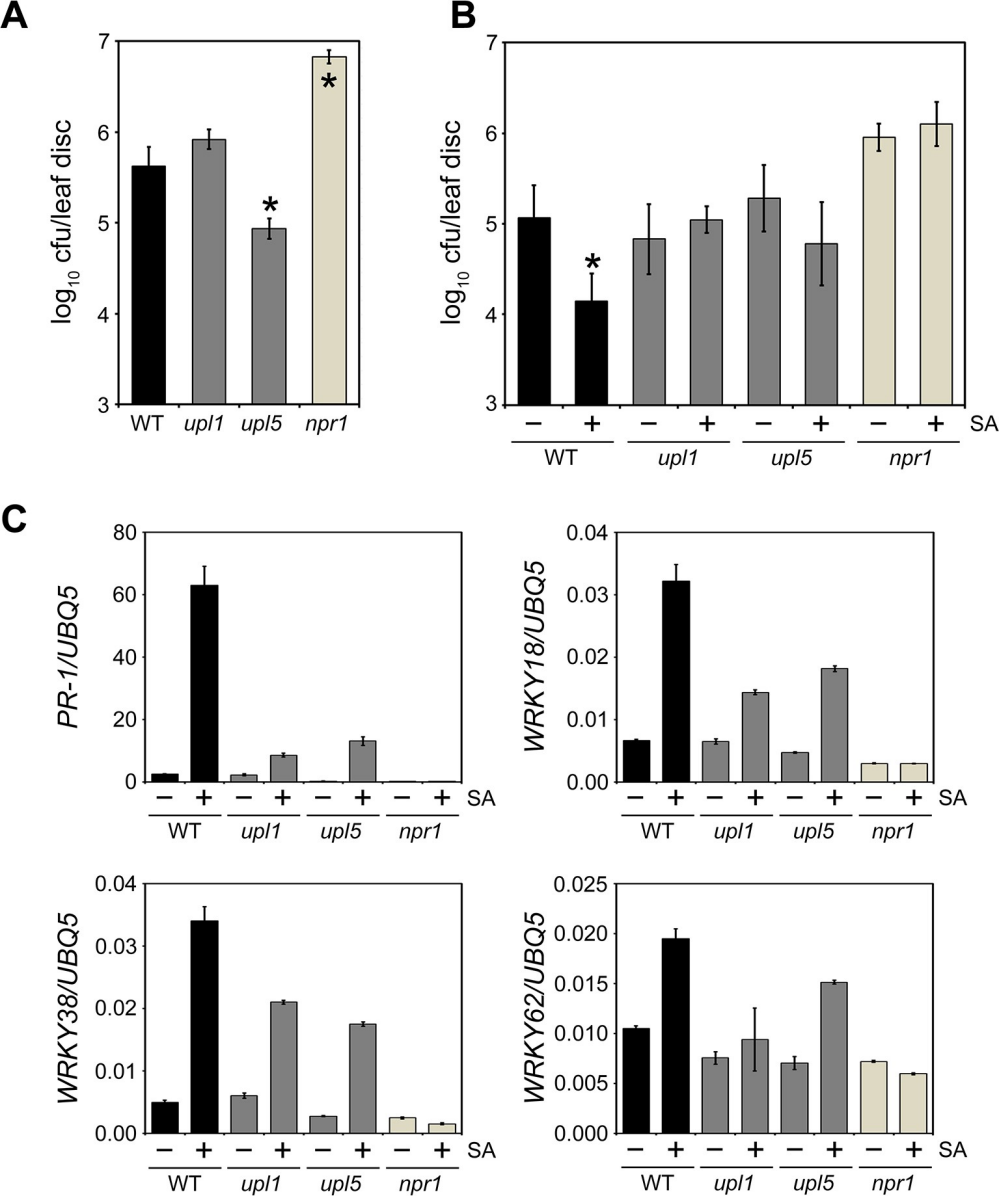
UPL1 and UPL5 control SA-mediated induced resistance responses. **(A)** Wild-type (WT), *upl1*, *upl5* and *npr1* plants were infected with *Psm* ES4326 (5 x 10^5^ cells) and pathogen growth (bottom panel) assessed after 5 days. Cfu, colony forming units. Error bars represent statistical 95% confidence limits (n = 8) and asterisks indicate statistically significant differences compared to WT (Tukey-Kramer ANOVA test; α = 0.05, n = 8). **(B)** Wild-type (WT), *upl1*, *upl5* and *npr1* plants were treated with 0.5 mM SA for 24 hours after which plants were infected with *Psm* ES4326 (5 x 10^6^ cells) and pathogen growth assessed after 4 days. Cfu, colony forming units. Error bars represent statistical 95% confidence limits (n = 8) and asterisks indicate statistically significant differences between mock (-) and SA (+) treatments for each genotype (Tukey-Kramer ANOVA test; α = 0.05, n = 8). **(C)** Adult wild-type (WT), *upl6*, *upl7* and *npr1* plants were treated with (+) or without (-) 0.5 mM SA for 24 hours. Expression of the immune marker genes *PR-1*, *WRKY18*, *WRKY38* and *WRKY62* was analysed by qPCR and normalised against constitutively expressed *UBQ5*. Error bars represent SD (n = 3).

Next we investigated if UPL ubiquitin ligases with ubiquitin-related domains were involved in orchestrating immune responses. UPL1 and UPL2 are closely related, containing both UBA and UIM signatures, whereas UPL5 harbours an ubiquitin domain (Fig 1). We selected knockout mutants for each (S1 Fig) and infected these plants with *Psm* ES4326. At a low infection dosage all three mutants exhibited resistance responses, whereas control *npr1* mutants showed the expected disease susceptible phenotype (Figs 3A and S2A). In some bioassays *upl5* allowed slightly lower growth of *Psm* ES4326 (Fig 3A), but this was inconsistent between assays and did not occur at higher inoculation dosages (Fig 3B). Therefore we conclude that *upl1*, *upl2* and *upl5* display relatively normal basal resistance responses.

To examine if these UPL ligases regulate induced resistance as activated by SA, plants were treated with SA prior to infection with *Psm* ES4326. While SA treatment induced immunity against this pathogen in wild-type and *upl2* plants, it failed to enhance resistance in both *upl1* and *upl5* mutants, which instead resembled the SA-insensitive *npr1* mutant in this respect (Figs 3B and S2B). To assess if UPL1 and UPL5 mediate SA signalling, we investigated SA-responsive immune gene expression. Treatment with SA induced strong, NPR1-dependent expression of *PR-1* and several *WRKY* genes in wild-type plants (Fig 3C). By contrast, activation of these genes was strongly reduced in both *upl1* and *upl5* mutant plants. Together these data indicate that UPL1 and UPL5 are positive regulators of SA-mediated gene expression and immunity.

### UPL3 regulates global SA-induced transcriptional reprogramming and immunity

**Fig 4:**
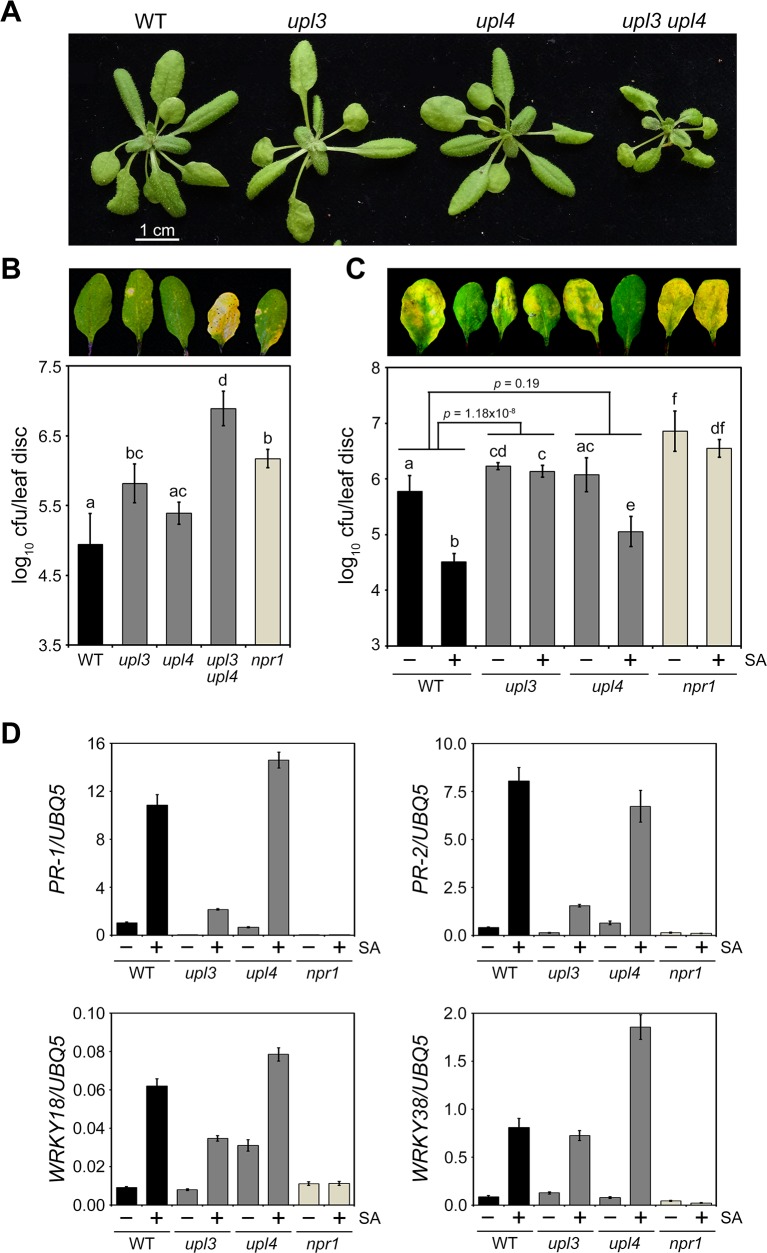
UPL3 and UPL4 control SA-mediated basal and induced resistance responses. **(A)** Morphological phenotypes of adult wild type (WT) and *upl3* or *upl4* single and double mutants. **(B)** Wild type (WT), *upl3* or *upl4* single and double mutants, and *npr1* plants were infected with *Psm* ES4326 (5 x 10^5^ cells) and disease symptoms (top panel) as well as pathogen growth (bottom panel) assessed after 4 days. Cfu, colony forming units. Error bars represent statistical 95% confidence limits (n = 8) and lowercase letters indicate statistically significant differences between genotypes (Tukey-Kramer ANOVA test; α = 0.05, n = 8). **(C)** Wild-type (WT), *upl3*, *upl4* and *npr1* plants were treated with 0.5 mM SA for 24 hours after which plants were infected with *Psm* ES4326 (5 x 10^6^ cells) and disease symptoms (top panel) as well as pathogen growth (bottom panel) assessed after 4 days. Cfu, colony forming units. Error bars represent statistical 95% confidence limits (n = 8) and lowercase letters indicate statistically significant differences between treatments and genotypes (Tukey-Kramer ANOVA test; α = 0.05, n = 8). Additionally, *p* values list statistical differences between the levels of SA-induced resistance between selected genotypes. **(D)** Adult wild-type (WT), *upl3*, *upl4* and *npr1* plants were treated with (+) or without (-) 0.5 mM SA for 24 hours. Expression of the immune marker genes *PR-1*, *PR-2*, *WRKY18* and *WRKY38* was analysed by qPCR and normalised against constitutively expressed *UBQ5*. Error bars represent SD (n = 3).

Similar to UPL1, the related UPL3 and UPL4 ubiquitin ligases contain domains with armadillo-type folds (Fig 1). Mutant *upl3* plants have previously been reported to exhibit aberrant leaf trichome morphology [36], but otherwise show normal growth and development (Figs 4A and S1). Similarly, *upl4* knockout mutants also exhibited normal growth (Fig 4A). Infection with a low dosage of *Psm* ES4326 revealed that compared to wild type, mutant *upl4* plants displayed normal disease resistance, while *upl3* mutants were more susceptible to this pathogen (Fig 4B). Because UPL3 and UPL4 are closely related, we generated *upl3 upl4* double mutants that showed reduced growth, early senescence and produced fewer seeds compared to either parent (Figs 4A and S3A). Infection of *upl3 upl4* double mutants resulted in striking leaf chlorosis and enhanced levels of *Psm* ES4326 growth (Fig 4B). These data indicate that UPL3 and UPL4 function additively in the regulation of plant growth and development, and positively modulate basal resistance.

Given the pleiotropic phenotypes of the *upl3 upl4* double mutant, we decided to continue with our investigation into the single mutants instead. Treatment with SA of mutant *upl4* plants induced resistance to *Psm* ES4326 to a similar extent as in wild type (Fig 4C). By contrast, *upl3* mutants resembled the SA-insensitive *npr1* mutant in that SA failed to induce resistance to *Psm* ES4326 (Fig 4C). This phenotype was observed in multiple mutant *upl3* alleles and constitutive expression of a transgene consisting of *Yellow Fluorescent Protein* fused to *UPL3* (*YFP-UPL3*) rescued SA-induced resistance in the *upl3* mutant background (S3B Fig). However, constitutively expressed *YFP-UPL3* did not rescue basal resistance to *Psm* ES4326, suggesting that dynamic *UPL3* expression or 5’ and 3’ untranslated regions, which were not included in our expression construct, may also play important gene regulatory roles. The immune phenotypes observed above agreed with the SA-responsive gene expression patterns we subsequently uncovered in the mutants. While *upl4* mutants showed predominantly wild type-like immune gene expression profiles in response to SA, mutant *upl3* plants failed to activate several immune marker genes (Fig 4D). Again this phenotype was observed in multiple mutant *upl3* alleles and constitutive expression of YFP-UPL3 restored SA-responsive *PR-1* gene expression in the *upl3* mutant background (S3C Fig).

**Fig 5:**
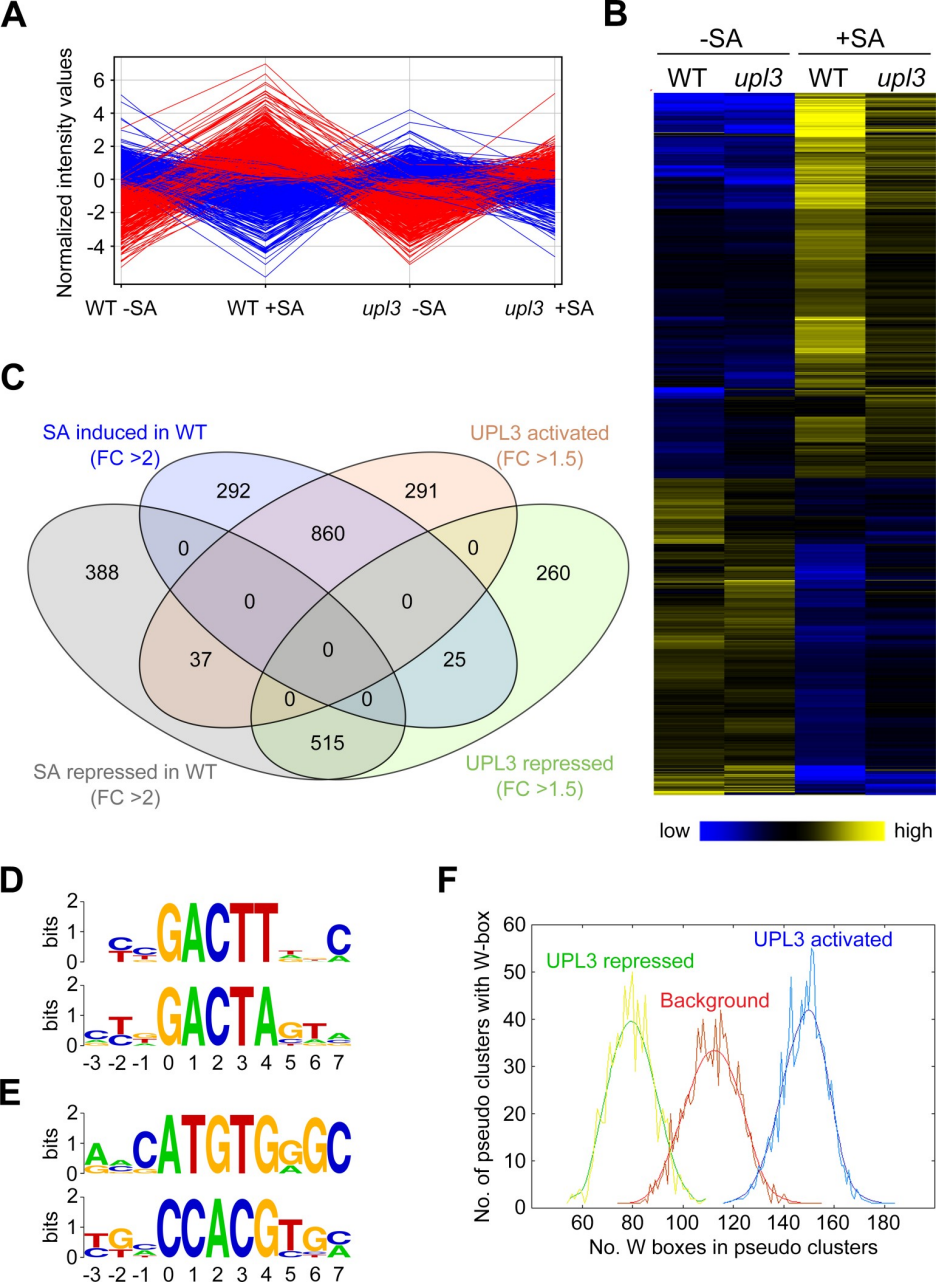
UPL3 amplifies SA-mediated transcriptional reprogramming. **(A)** Profile plot of SA-induced and repressed genes in wild-type (WT) and mutant *upl3* plants. Adult WT and *upl3* plants were treated with water or SA, mRNA extracted and analysed by RNA Seq. Up- (red) and downregulated (blue) genes with fold change of ≥ 2 in WT (ANOVA; *p = 0*.*05*, n = 3) are shown. **(B)** Heat map of SA-induced and repressed genes in wild-type (WT) and mutant *upl3* plants. Up- (yellow) and downregulated (blue) genes with fold change of ≥ 2 in WT (ANOVA; *p = 0*.*05*, n = 3) are shown. **(C)** Venn diagram illustrating that UPL3 significantly amplifies the up- or downregulation of SA-regulated genes. The diagram compares gene expression from SA-treated WT to SA-treated *upl3* mutants. Only SA-regulated genes that showed fold changes of ≥ 2 in WT (ANOVA; *p = 0*.*05*, n = 3) were included in the analyses with SA-induced and SA-repressed genes shown in blue and grey ovals, respectively. Pink and green ovals indicate genes that exhibited ≥ 1.5 fold downregulation (*i*.*e*. UPL3-dependent activation) or upregulation (*i*.*e*. UPL3-dependent repression) in *upl3* compared to WT (ANOVA; *p = 0*.*05*, n = 3). **(D, E)** Overrepresented octamer sequences in UPL3 activated (D) and UPL3 repressed (E) gene promoters were aligned and subjected to Weblogo (http://weblogo.berkeley.edu/logo.cgi). The Y-axis indicates the relative frequency and sequence conservation. **(F)** W-box representation analyses of the top 284 gene promoters (1000 bp upstream of transcriptional start site) downregulated in *upl3* compared to WT. POBO analyses showed that compared to 1000 randomly selected promoters (background), the W-box is highly overrepresented in UPL3 activated genes, while underrepresented in UPL3 repressed genes.

To explore if reduced SA-responsive marker gene expression was a transcriptome-wide effect, we performed an RNA Seq experiment on SA-treated wild-type and mutant *upl3* plants. SA treatment resulted in differential expression of 2,117 genes (≥ 2 fold, *p* = 0.05) of which 1,177 were up- and 940 downregulated after 24 hours. Although some changes were detected between control-treated wild-type and *upl3* plants, much larger differential gene expression changes became apparent after SA treatment (Fig 5A). Differences in gene expression were mostly in amplitude with less dramatic activation or repression observed in *upl3* mutants compared to the wild type (Figs 5A, 5B and S4 and S1 Table). Indeed, of the 1,177 genes activated by SA in the wild type, 860 were expressed at least 1.5-fold lower in *upl3* mutants (Figs 5C and S5A). Conversely, 515 of 940 SA-repressed genes were down regulated at least 1.5-fold less in *upl3* mutants (Figs 5C and S5B). These data suggest that UPL3 acts to amplify SA-responsive gene expression changes. To identify the binding sites of potential transcription factors on which UPL3 may act, we performed promoter motif analyses on differentially expressed SA-responsive genes. Analyses of SA-induced UPL3-dependent promoters revealed they are enriched with variants of the immune-related W-box motif (Fig 5D), while promoters that were suppressed by SA in a UPL3-dependent manner contained variants of the developmental E-box motif (Fig 5E). The W-box motif binds WRKY transcription factors, several of which are indispensable for the full activation of SA-dependent gene expression and immunity [23, 28]. As the W-box is pervasive in SA-responsive genes [23, 38] and was highly enriched in UPL3 activated but not in UPL3 repressed genes (Fig 5F), our findings indicate that UPL3 acts as a genome-wide amplifier of SA-responsive transcriptional reprogramming and establishment of immunity.

### Proteasome-associated ubiquitin ligase activity is UPL3 dependent and increases cellular polyubiquitination levels

**Fig 6:**
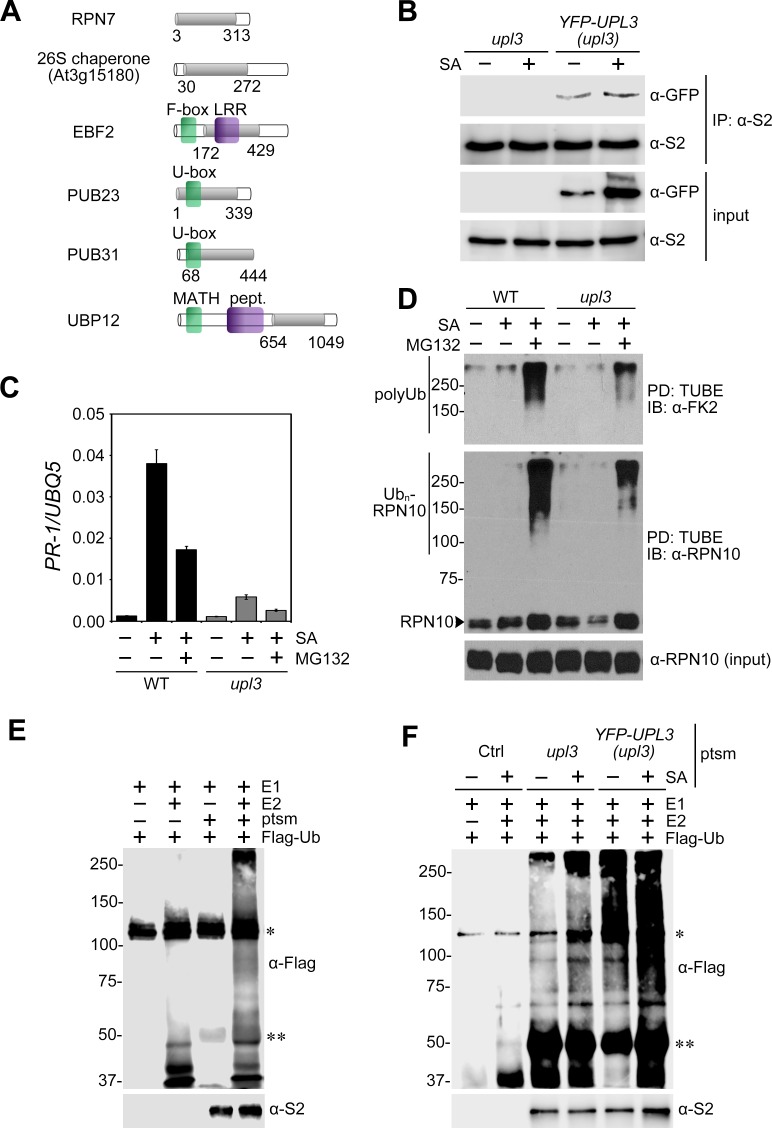
UPL3 generates ubiquitin chains at the proteasome. **(A)** Yeast two-hybrid interactors of UPL3. The N-terminal 670 amino acids of UPL3 were used as bait to identify the indicated interacting prey proteins. Protein domains are indicated in green and purple (LRR, leucine-rich repeat; MATH, Meprin and TRAF homology domain; pept., peptidase), whereas amino acid sequence shared by all prey fragments are shaded in grey. **(B)** UPL3 physically associates with the proteasome *in planta*. Adult plants of the indicated genotypes were treated for 24 hours with 0.5 mM SA. Proteasomes were immunoprecipitated (IP) with an antibody against the S2 subunit. Inputs and immunoprecipitated proteins were analysed by western blotting using antibodies against GFP and S2. **(C)** Suppression of SA-induced gene expression in *upl3* mutants resembles proteasome inhibition. Wild-type (WT) and *upl3* plants were treated with (+) or without (-) 0.5 mM SA and 100 μM MG132 for 6 hours. The expression of *PR-1* was analysed by qPCR and normalised against constitutively expressed *UBQ5*. Error bars represent SD (n = 3). **(D)** UPL3 facilitates total cellular polyubiquitination levels. Wild-type (WT) and *upl3* plants were treated with (+) or without (-) 0.5 mM SA and 100 μM MG132 for 6 hours. Ubiquitinated proteins were pulled down (PD) using GST-tagged tandem ubiquitin binding entities (TUBE). Total (input) and pulled down proteins were analysed by immunoblotting (IB) using antibodies against ubiquitin conjugates (polyUb, α-FK2) and RPN10. Both unmodified RPN10 and polyubiquitinated RPN10 (RPN10-Ub_n_) are indicated. **(E)** Proteasomes harbour E3 ligase activity. Proteasomes (ptsm) were immunoprecipitated with an anti-S2 antibody from WT plants that were treated for 6 hours with 0.5 mM SA. Proteasomes were then incubated with E1 enzyme, E2 enzyme, Flag-ubiquitin (Flag-Ub) and ATP. Mock immunoprecipitations in absence of anti-S2 antibody were performed as controls. Reactions products were detected by western blotting using anti-Flag and anti-S2 antibodies. *, self-ubiquitinated E1; **, cross-reacting IgG heavy chain. **(F)** UPL3 is responsible for proteasome-associated E3 ligase activity. Experiment was performed as in (E) except proteasomes (ptsm) were immunoprecipitated from indicated genotypes that were treated for 24 hours with or without 0.5 mM SA. Mock immunoprecipitations in absence of anti-S2 antibody were performed as controls (Ctrl).

To understand how UPL3 might function as a general transcriptional amplifier for SA-responsive genes, we performed a yeast two-hybrid screen for interactors. Because the N-terminus of UPL3 contains armadillo repeats (Fig 1) that are thought to provide a large surface for protein-protein interactions [39], we used the N-terminal 670 amino acids as bait. In addition to self-interaction, we identified six components related to the ubiquitin-26S proteasome system (Fig 6A, S2 Table). These included the non-ATPase regulatory subunit RPN7 which forms part of the 19S regulatory particle, as well as the armadillo-repeat superfamily protein At3g15180 that contains a domain (InterPro:IPR019538) found in proteasomal chaperones involved in assembly of the proteasome [40]. Moreover, we identified three E3 ubiquitin ligases: (*i*) the F-box protein EBF2 which is part of an SCF^EBF1/2^ ubiquitin ligase that targets the ethylene-responsive transcription factor EIN3 for proteasome-mediated degradation [41, 42]; (*ii*) the U-box type E3 ligase PUB23 that has been implicated in plant immunity, interacts with and ubiquitinates the 19S proteasome regulatory particle subunit RPN6 [43, 44]; and (*iii*) the U-box type E3 ligase PUB31 that is involved in abiotic stress tolerance [45]. Finally, UPL3 was found to interact with UBP12, a deubiquitinase of the proteasome pathway that negatively regulates immunity [46].

In agreement with these protein-protein interactions, UPL3 was found previously to co-purify with a pathogen effector that targets proteasomes [47], suggesting UPL3 may physically associate with proteasomes. Indeed, pull down of the proteasomal subunit S2 revealed that YFP-UPL3 co-immunoprecipitated with proteasomes largely independent of SA treatment (Fig 6B). Next we considered how physical association with the proteasome allows UPL3 to function as an amplifier of the SA-responsive transcriptome. SA-responsive gene expression strongly depends on the function of the 26S proteasome [8, 28]. Indeed, reduced activation of SA-responsive immune genes in *upl3* mutants resembled the effect of pharmacological inhibition of the proteasome with the proteasomal inhibitor MG132 in SA-treated wild-type plants (Fig 6C). Given the interconnection between UPL3 and multiple components of the ubiquitin-26S proteasome system, including the 19S subunit, we considered that UPL3 may regulate gene expression by altering total cellular ubiquitination levels. Therefore we treated wild-type and *upl3* plants with SA and/or MG132 and pulled down ubiquitinated proteins. Figs 6D and S6 show that compared to wild type, *upl3* mutants exhibited markedly reduced levels of total cellular polyubiquitination. Moreover, ubiquitination of RPN10, a substrate of many different ubiquitin ligase types [48], was also reduced. This remarkable phenotype suggests that UPL3 promotes polyubiquitination of either a small group of heavily ubiquitinated proteins or an extraordinary wide range of substrates.

Our findings suggest that UPL3 may aid the proteasome to reinforce polyubiquitination of its substrates upon their arrival. To explore if plant proteasomes harbour E3 ligase activity immunopurified proteasomes were incubated with E1 and E2 enzymes, Flag-ubiquitin and ATP. Under these conditions proteasomes readily converted free ubiquitin into conjugates (Fig 6E). To investigate if this proteasome-associated E3 ligase activity was dependent on UPL3, we repeated the assay by comparing proteasomes from *upl3* mutants with or without expression of *YFP-UPL3*. Proteasomes from water-treated *YFP-UPL3* (in *upl3*) plants formed polyubiquitin conjugates and this activity was stimulated by prior treatment with SA (Fig 6F). By contrast, proteasomes from both water- and SA-treated *upl3* mutants exhibited markedly reduced formation of ubiquitin conjugates, demonstrating that proteasome-associated ubiquitin ligase activity was largely UPL3 dependent. Taken together our findings suggest UPL3-dependent proteasome-associated ubiquitin ligase activity is necessary for SA-responsive transcriptional reprogramming and immunity.

## Discussion

The ubiquitin-26S proteasome system plays indispensable roles in transcriptional regulation of plant immune genes but how substrates are processed upon arrival at the proteasome remained unclear. Here we demonstrated that members of the HECT-domain family of UPL ubiquitin ligases play an important role in SA-dependent transcriptional responses and immunity. In particular we report that proteasomes harbour UPL3-dependent ubiquitin ligase activity that was necessary for total cellular substrate polyubiquitination as well as SA-responsive transcriptional reprogramming and immunity.

Our findings show that UPL1, UPL3, UPL4 and UPL5 function as important regulators of SA-responsive gene expression and immunity (Figs 3, 4 and 5). Previous work has found that UPL members play roles in developmental gene expression programmes. UPL3 has been reported to regulate trichome branching by targeting for proteasomal degradation the transcription factors GLABROUS 3 (GL3) and ENHANCER OF GL3 (EGL3), which control trichome development and flavonoid metabolism [36, 49]. UPL5 was identified as an interactor of WRKY53, a transcription factor that promotes leaf senescence [50]. *In vitro* and *in vivo* analyses indicated that UPL5 ubiquitinated WRKY53 and targeted it for degradation. Consequently, mutant *upl5* plants displayed enhanced expression of a WRKY53-responsive senescence marker gene and accelerated appearance of senescing leaves [51]. Interestingly, WRKY53 is not only a regulator of developmental responses; it was also identified as a regulator of SA-dependent plant immunity. *WRKY53* gene expression is SA inducible and a direct transcriptional target of the master immune coactivator NPR1. Mutation of *WRKY53* together with *WRKY70*, whose expression is highly correlated with *WRKY53*, resulted in susceptibility to *Psm* ES4326 [23]. Therefore it is plausible that UPLs also regulate the stability of WRKY transcription factors during activation of plant immunity. Indeed, transcriptomic analyses of *upl3* mutants indicated that the W-box to which WRKY transcription factors bind, was highly overrepresented in SA-induced, UPL3-dependent gene promoters (Fig 5). UPLs could remove repressors such as WRKY58 from immune-responsive promoters or facilitate the turnover of WRKY activators whose transcriptional activity may require instability akin to NPR1 coactivator [8, 23, 28, 52]. In this respect, it is worth noting that the broad impact of UPL3 on the SA-responsive transcriptome resembles that of WRKY18, which functions as an auxiliary amplifier of SA-responsive gene expression [23].

Mutation of *UPL3* had a remarkable impact on total cellular polyubiquitination levels, a phenotype rarely observed for E3 ubiquitin ligase mutants. So how could UPL3 have such a large effect on the cellular accumulation of so many polyubiquitin conjugates? Yeast two-hybrid assays indicated that UPL3 may associate with the 19S regulatory particle of the proteasome (Fig 6A, S2 Table) and *in planta* YFP-UPL3 co-immunoprecipitated with proteasomes (Fig 6B). We show that this interaction was responsible for proteasome-associated E3 ligase activity (Fig 6). Several proteasome-associated ubiquitin ligases have been described and consequently it has been proposed that instead of regarding substrate ubiquitination and delivery to the proteasome as separate steps, these two steps may in fact be coupled for some substrates [32]. Coupling of ubiquitination to degradation may enhance substrate affinity for proteasome receptors or prevent substrate deubiquitination. Thus, proteasome-associated ubiquitin ligases could have large substrate repertoires. Indeed, the yeast ubiquitin ligase HUL5 and its mammalian homologue KIAA10 are abundantly associated with the proteasome 19S regulatory subcomplex and show high sequence similarity to Arabidopsis UPL3 [30, 31, 53]. Similar to knock-out of *UPL3* reported here, deletion of *HUL5* led to a total cellular reduction in polyubiquitinated substrates [31]. HUL5 appears to indiscriminately amplify the degradation of substrates by elongating their ubiquitin chains, an activity that is not typical for an E3 ubiquitin ligase. Whereas most E3 enzymes have specific substrate targets, E4 enzymes are thought to extend existing ubiquitin chains without much apparent specificity [54]. Instead, their co-location with protein complexes such as the proteasome may provide substrate specificity [31, 55]. Thus, we propose that similar to the E4 enzyme activity of HUL5, UPL3 may also function to elongate ubiquitin chains of proteasome-bound substrates. The importance of this activity was previously demonstrated by substrate stalling and incomplete degradation by proteasomes in *hul5Δ* mutant yeast and in human cells by knocking down the orthologue UBE3C, indicating that ubiquitin chain elongation is necessary for processive degradation of substrates [34, 35]. Likewise, proteasomal association of another yeast HECT-type ubiquitin ligase, UFD4, which also shows high sequence similarity to UPL3, including Armadillo repeats, was found to be necessary for complete substrate degradation [56]. Proteasomal stalling or incomplete degradation of immune-related transcriptional regulators could explain the immune compromised phenotypes of mutant *upl3* plants.

To date a number of prototypical E3 ubiquitin ligases have been found to also associate with the proteasome, albeit in lower abundance than for example HUL5. Remarkably, core and variable subunits of the modular SCF ubiquitin ligase also bind the proteasome [32, 57]. In Arabidopsis the major developmental SCF ligase substrate adapters UFO, COI1 and TIR1 associate with the proteasome [58], further supporting the notion that ubiquitination of substrates and their proteasomal delivery may be directly coupled processes. Here we report that UPL3 may associate with E3 ligases, including ethylene-responsive SCF^EBF2^ as well as PUB23 and PUB31 U-box type E3 ligases (Fig 6A, S2 Table). SCF^EBF2^ targets for proteasomal degradation the indispensable ethylene-responsive EIN3 transcription factor that cross-regulates SA biosynthesis and SA-responsive genes [59, 60], while PUB23, together with its homologues PUB22 and PUB24, mediates pattern recognition receptor-mediated immune signalling by targeting exocytosis regulators [43, 61]. Thus, it is plausible that in addition to regulating proteasomal degradation of substrates from the SA signalling pathway, UPL3 may also control immunity by cooperating with E3 ligases from other immune-associated pathways. Such cooperation between E4 ligase-like activities and E3 ligases has been suggested previously. In yeast the RING-type E3 ligase Ubr1, which targets N-end rule pathway substrates for proteasomal degradation, physically interacted with UFD4, resulting in the formation of longer substrate-attached polyubiquitin chains [62]. This and an additional report [62] of interaction between HECT-type and other E3 ligases suggest that ubiquitin ligase pairing at the proteasome facilitates processive ubiquitination and degradation of substrates.

In conclusion, our findings implicate proteasome-associated HECT-type ubiquitin ligases in the control of plant immune signalling by facilitating substrate polyubiquitination and proteasomal processivity. We reveal this unexpected E4 ligase-like activity plays important roles in the genome-wide amplification of SA-responsive gene transcription and is indispensable for establishment of immunity.

## Materials and methods

### Plant lines, chemical induction and pathogen infection

*Arabidopsis thaliana* wild-type Col-0, transgenic and mutant plants were sown on soil and grown under a 16/8 hr light/dark regime. After 10–12 days seedlings were separated and transferred to larger pots and grown for an additional 2.5–3 weeks. Mutant *upl1-1* (SALK_063972), *upl2-2* (SALK_008974), *upl3-2* (SAIL_339_F05) [36], *upl3-4* (SALK_035524), *upl4-1* (SALK_091246), *upl5-1* (SALK_116446), *upl6-1* (SALK_055609), *upl7-1* (SALK_119373) were isolated from the SALK and SAIL collections [63, 64] and the *npr1-1* mutation has been described previously [24]. Double mutants were created by crossing *upl3-4* with a second *UPL4* knockout mutant allele, *upl4-2* (SALK_040984), while *upl6* was crossed with a second *UPL7* knockout mutant allele, *upl7-2* (SAIL_403_A11). According to the manufacturer’s instructions the coding sequence of *UPL3* (At4g38600) was cloned into pCR8/GW/TOPO (Thermo-Fisher Scientific) and recombined with YFP-containing pEarleyGate 104 (Earley et al., 2006) using LR clonase (Life Technologies) to generate the *35S*::*YFP-UPL3* transgene. The *35S*:: *YFP-UPL3* vector was transferred into *Agrobacterium tumefaciens* strain GV3101 (pMP90) using a freeze-thaw method and subsequently transformed into *upl3-4* plants by floral dipping [65]. Transgenic plants were selected on soil by repeatedly spraying glufosinate ammonium.

*Psm* ES4326 was grown overnight in liquid LB medium supplemented with 10 mM MgSO_4_. Bacterial cells were collected by centrifugation, diluted to the appropriate concentrations and pressure-infiltrated into leaves. *In planta* bacterial growth was determined 4–5 days after infection by spreading serial dilutions of leaf extracts on LB plates supplemented with streptomycin (100 μg/ml), 10 mM MgSO_4_ and 50 μM cycloheximide. To test induced resistance adult plants were sprayed 24 hours prior to pathogen infiltration with water or 0.5 mM SA (sodium salicylate, Sigma-Aldrich #S3007) until the leaves were extensively covered with fine droplets. For induction of immune genes and protein analyses, 4-week old soil-grown plants were sprayed with water or 0.5 mM SA until the leaves were extensively covered with fine droplets. Alternatively, 12-day-old MS-grown seedlings were submerged in 6-well plates containing 10 ml (per well) of water supplemented with or without 0.5 mM SA for 6 hours. For proteasome inhibition experiments, seedlings were submerged in solutions containing vehicle (DMSO), 0.5 mM SA and vehicle, or 0.5 mM SA and 100 μM MG132 for 6 hours.

### Gene expression analysis

RNA extractions and cDNA synthesis were performed as described [28]. Quantitative qPCR was carried out on 20-times diluted cDNA using Power SYBR Green (Life Technologies) and gene-specific primers on a StepOne Plus Real Time PCR system (Life Technologies).

For RNA Seq analyses, RNA was extracted from biological triplicate samples as described [28] and further purified using an RNeasy Mini Kit (Qiagen) according to the manufacturer’s instructions. qPCR was carried out to confirm appropriate induction of SA-responsive marker genes. RNA was then quantified and submitted to GATC Biotech (Constance, Germany) for RNA sequencing. The RNA Seq reads were aligned to the *Arabidopsis thaliana* TAIR10 genome using Bowtie. TopHat identified potential exon-exon splice junctions of the initial alignment. Strand NGS software in RNA Seq workflow was used to quantify transcripts. Raw counts were normalised using DESeq with baseline transformation to the median of all samples. Data were then expressed as normalised signal values (*i*.*e*. log_2_[RPKM] where RPKM is read count per kilobase of exon model per million reads) for all statistical tests and plotting. RNA-seq data have been deposited in the ArrayExpress database at EMBL-EBI (www.ebi.ac.uk/arrayexpress) under accession number E-MTAB-7374.

Extraction of overrepresented octamer sequences was performed as previously reported [66] on the top 281 and 292 differentially expressed UPL3-activated and UPL3-repressed gene promoters, respectively. The enriched octamers were aligned according to a conserved pentamer sequence, followed by analysis using Weblogo version 2.8.2 (http://weblogo.berkeley.edu/). Additionally, promoters were analysed for statistical over- or underrepresentation of the W-box using POBO [67].

### Identification of UPL3 interactors by yeast two-hybrid screening

Yeast two-hybrid screening and data analyses were performed by Hybrigenics Services (Paris, France). Amino acids 1–670 of UPL3 were cloned into vector pB29 (N-UPL3-LexA-C fusion) and screened against a prey library derived from RNA extracted from *Arabidopsis thaliana* rosettes infected either with virulent *P*. *syringae* pv. tomato DC3000 or with an avirulent strain expressing AvrRpt2. A total of 65.2 million interactions were analysed and 353 positive clones sequenced. Interactions were categorised by confidence scores that are based on a statistical model of the competition for bait-binding between fragments [68, 69].

### Protein analyses

For co-immunoprecipitation experiments, tissue was pulverised in liquid nitrogen and protein extracted in 2 volumes of proteasome extraction buffer containing 125 mM Tris-HCl (pH7.7), 0.25 mM EDTA, 2.5 mM MgCl_2_, 5% glycerol, 5 mM ATP, and protease inhibitors (50 μg/mL N-p-Tosyl-L-phenylalanine chloromethyl ketone (TPCK), 50 μg/mL Nα-Tosyl-L-lysine chloromethyl ketone hydrochloride (TLCK), 0.6 mM phenylmethylsulfonyl fluoride (PMSF)). Protein extracts were centrifuged (17,000 *g*, 20 min. at 4°C), supernatants filtered through 0.22 μM syringe filters and incubated for 2 hours at 4°C with anti-proteasome S2 antibody (Abcam, ab98865 at ratio 1:250). Next, protein A-agarose was added (20 μl/ml) and incubated with gentle rocking for another hour. Agarose beads were collected by brief centrifugation and washed 5 times with extraction buffer. Bound proteins were eluted by incubation in SDS sample buffer supplemented with 50 mM dithiothreitol (DTT) for 5 min. at 95°C.

For analyses of cellular polyubiquitin conjugate levels, twelve-day-old seedlings were placed in solutions containing vehicle (DMSO), 0.5 mM SA and vehicle, or 0.5 mM SA and 100 μM MG132 for 6 hours. Tissue was then blotted dry and pulverised in liquid nitrogen. Protein was extracted in two volumes of extraction buffer, consisting of phosphate buffered saline supplemented with 1% Triton X-100, 10 mM N-ethylmaleimide, phosphatase inhibitor cocktail 3 (Sigma-Aldrich), protease inhibitor cocktail [50 μg/mL N-p-Tosyl-L-phenylalanine chloromethyl ketone (TPCK), 50 μg/mL Nα-Tosyl-L-lysine chloromethyl ketone hydrochloride (TLCK), 0.6 mM phenylmethylsulfonyl fluoride (PMSF)], and 0.2 mg/ml recombinant GST-tagged tandem ubiquitin binding entities (TUBE) [70]. Protein extracts were centrifuged (17,000 *g*, 20 min. at 4°C), supernatants filtered through 0.22 μM syringe filters and incubated overnight at 4°C with 50 μl/ml of packed Protino Glutathione Agarose 4B (Machery Nagel). Agarose was washed 5 times with extraction buffer and bound proteins eluted by incubation in SDS sample buffer supplemented with 50 mM dithiothreitol (DTT) for 10 min. at 80°C.

Proteasomal E3 ligase activity was assessed by extracting protein from liquid nitrogen pulverised tissue in 2 volumes of proteasome extraction buffer containing 125 mM Tris-HCl (pH7.7), 0.25 mM EDTA, 2.5 mM MgCl_2_, 5% glycerol, 5 mM ATP, and protease inhibitors (50 μg/mL N-p-Tosyl-L-phenylalanine chloromethyl ketone (TPCK), 50 μg/mL Nα-Tosyl-L-lysine chloromethyl ketone hydrochloride (TLCK), 0.6 mM phenylmethylsulfonyl fluoride (PMSF)). Protein extracts were centrifuged (17,000 *g*, 20 min. at 4°C), supernatants filtered through 0.22 μM syringe filters and incubated overnight at 4°C with anti-proteasome S2 antibody (Abcam, ab98865 at ratio 1:250). The next day extracts were centrifuged (17,000 *g*, 10 min. at 4°C) and supernatants collected. Protein A-agarose was then added (20 μl/ml) and incubated with gentle rocking for one hour. Agarose beads were collected by brief centrifugation and washed 3 times with proteasome extraction buffer. Subsequently, agarose beads were incubated for 18 hours with gentle shaking at 30°C in 80 μl reaction buffer (125 mM Tris-HCl (pH7.7), 0.25 mM EDTA, 2.5 mM MgCl_2_, 5 mM ATP, 1 mM DTT, 10 μM NSC632836 deubiquitinase inhibitor) supplemented with, human recombinant E1 enzyme (0.2 or 0.4 μg, BioVision), recombinant E2 enzyme UbcH5c (0.2 μg, Ubiquigent), and recombinant human Flag-ubiquitin (10 μg, Boston Biochem). Agarose beads were eluted by incubation in SDS sample buffer supplemented with 50 mM dithiothreitol (DTT) for 10 min. at 80°C.

All proteins were analysed by SDS-PAGE followed by western blotting using anti-ubiquitin (anti-ubiquitinylated proteins clone FK2, Merck), anti-RPN10 (polyclonal antibody against Arabidopsis RPN10, Abcam), anti-proteasome S2 (polyclonal antibody against full-length Arabidopsis S2, Abcam), anti-Flag (monoclonal anti-Flag M2 antibody, Sigma) and anti-GFP (mixture of monoclonal antibodies from clones 7.1 and 13.1, Roche) antibodies.

## Supporting information

S1 Fig Expression analyses of T-DNA knockout mutants for *UPL* genes. The expression of *UPL* genes was analysed by qPCR in wild-type (WT) and indicated *upl* mutant alleles.
(TIF)

S2 Fig Mutant *upl2* plants exhibit normal SA-dependent immunity. **(A)** Wild type (WT), *upl2* and *npr1* plants were infected with *Psm* ES4326 (5 x 10^5^ cells) and pathogen growth assessed after 4 days. Cfu, colony forming units. Error bars represent statistical 95% confidence limits (n = 8) and asterisks indicate statistically significant differences compared to WT (Tukey-Kramer ANOVA test; α = 0.05, n = 8).
**(B)** Wild type (WT), *upl2* and *npr1* plants were treated with 0.5 mM SA for 24 hours after which plants were infected with *Psm* ES4326 (5 x 10^6^ cells) and pathogen growth assessed after 4 days. Cfu, colony forming units. Error bars represent statistical 95% confidence limits (n = 8) and asterisks indicate statistically significant differences between mock (-) and SA (+) treatments for each genotype (Tukey-Kramer ANOVA test; α = 0.05, n = 8).
(TIF)

S3 Fig Allelic and complementation analyses for UPL3 and UPL4. **(A)** Seeds per silique in adult WT, *upl3* and *upl4* single, and *upl3 upl4* double mutants. Error bars represent SD (n = 10).
**(B)** Wild-type (WT), *upl3-2*, *upl3-4*, *35S*::*YFP-UPL3 (in upl3-4)* and *npr1* plants were treated with 0.5 mM SA for 24 hours after which plants were infected with *Psm* ES4326 (5 x 10^6^ cells) and disease symptoms (top panel) as well as pathogen growth (bottom panel) assessed after 3 days. Cfu, colony forming units. Error bars represent statistical 95% confidence limits (n = 8) and asterisks indicate statistically significant differences between mock (-) and SA (+) treatments for each genotype (Tukey-Kramer ANOVA test; α = 0.05, n = 8).
**(C)** Adult wild-type (WT), *upl3-2*, *upl3-4*, *35S*::*YFP-UPL3 (in upl3-4)* and *npr1* plants were treated with (+) or without (-) 0.5 mM SA for 24 hours. Expression of the immune marker genes *PR-1* was analysed by qPCR and normalised against constitutively expressed *UBQ5*. Error bars represent SD (n = 3).
(TIF)

S4 Fig qPCR validation of *PR* genes from RNA Seq samples. *PR* gene expression in adult WT and *upl3* plants treated for 24 hours with or without 0.5 mM SA was analysed by qPCR (left panels) and by RNA Seq (right panels). Error bars represent SD (n = 3).
(TIF)

S5 Fig UPL3 amplifies SA-mediated transcriptional reprogramming. **(A)** Heat map of 860 SA-induced genes from Venn diagram in Fig 5C for wild-type (WT) and mutant *upl3* plants. Highly expressed genes are shown in yellow whereas lowly expressed genes are shown in blue.
**(B)** Heat map of 515 SA-repressed genes from Venn diagram in Fig 5C for wild-type (WT) and mutant *upl3* plants. Highly expressed genes are shown in yellow whereas lowly expressed genes are shown in blue.
(TIF)

S6 Fig UPL3 facilitates total cellular polyubiquitination levels. Long exposure of anti-ubiquitin blot shown in Fig 6D. Wild-type (WT) and *upl3* plants were treated with (+) or without (-) 0.5 mM SA and 100 μM MG132 for 6 hours. Ubiquitinated proteins were pulled down (PD) using GST-tagged tandem ubiquitin binding entities (TUBE). Total (input) and pulled down proteins were analysed by immunoblotting (IB) using antibodies against ubiquitin conjugates (polyUb, α-FK2) and RPN10.
(TIF)

S1 Table SA-induced differentially expressed genes in wild-type versus *upl3* plants. List of genes differentially expressed between different condition (*i*.*e*. genotype and treatment) pairs that exhibited at least 1.5 fold down- or upregulation in one at least one comparison (ANOVA; *p = 0*.*05*, n = 3).
(XLSX)

S2 Table Yeast two-hybrid interactors of UPL3. List of yeast two-hybrid prey proteins with recovered sequences and confidence scores identified when using the UPL3 N-terminal domain (amino acids 1–670) as bait.
(XLSX)
