# Supplementary figures and images for: Proteasome-associated HECT-type ubiquitin ligase activity is required for plant immunity
Source: PLoS Pathog. 2018 Nov 20;14(11):e1007447. doi: 10.1371/journal.ppat.1007447 (PMC6286022; doi:10.1371/journal.ppat.1007447)

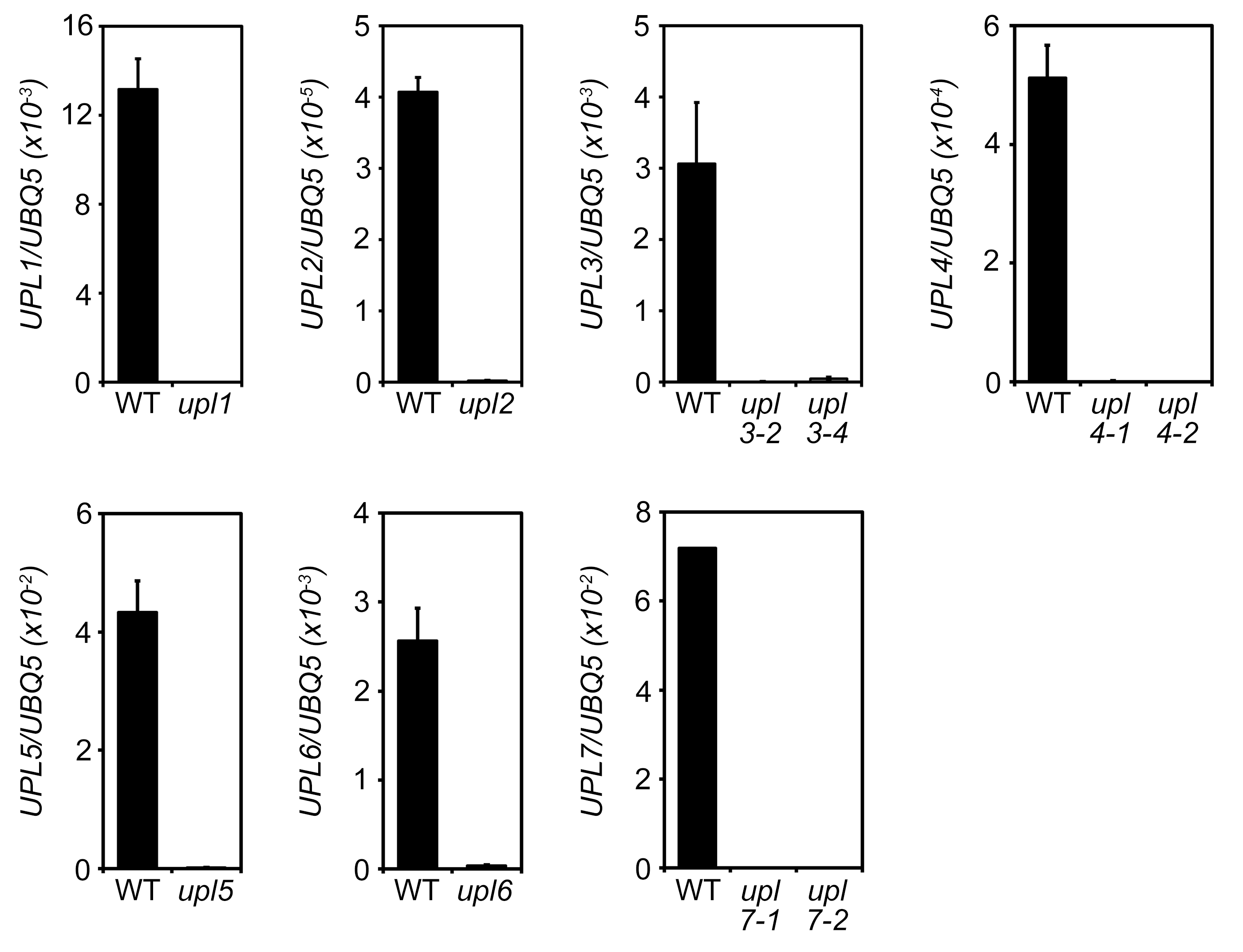

Supplement: S1 Fig — The expression of UPL genes was analysed by qPCR in wild-type (WT) and indicated upl mutant alleles. (TIF) [file ppat.1007447.s001.tif]

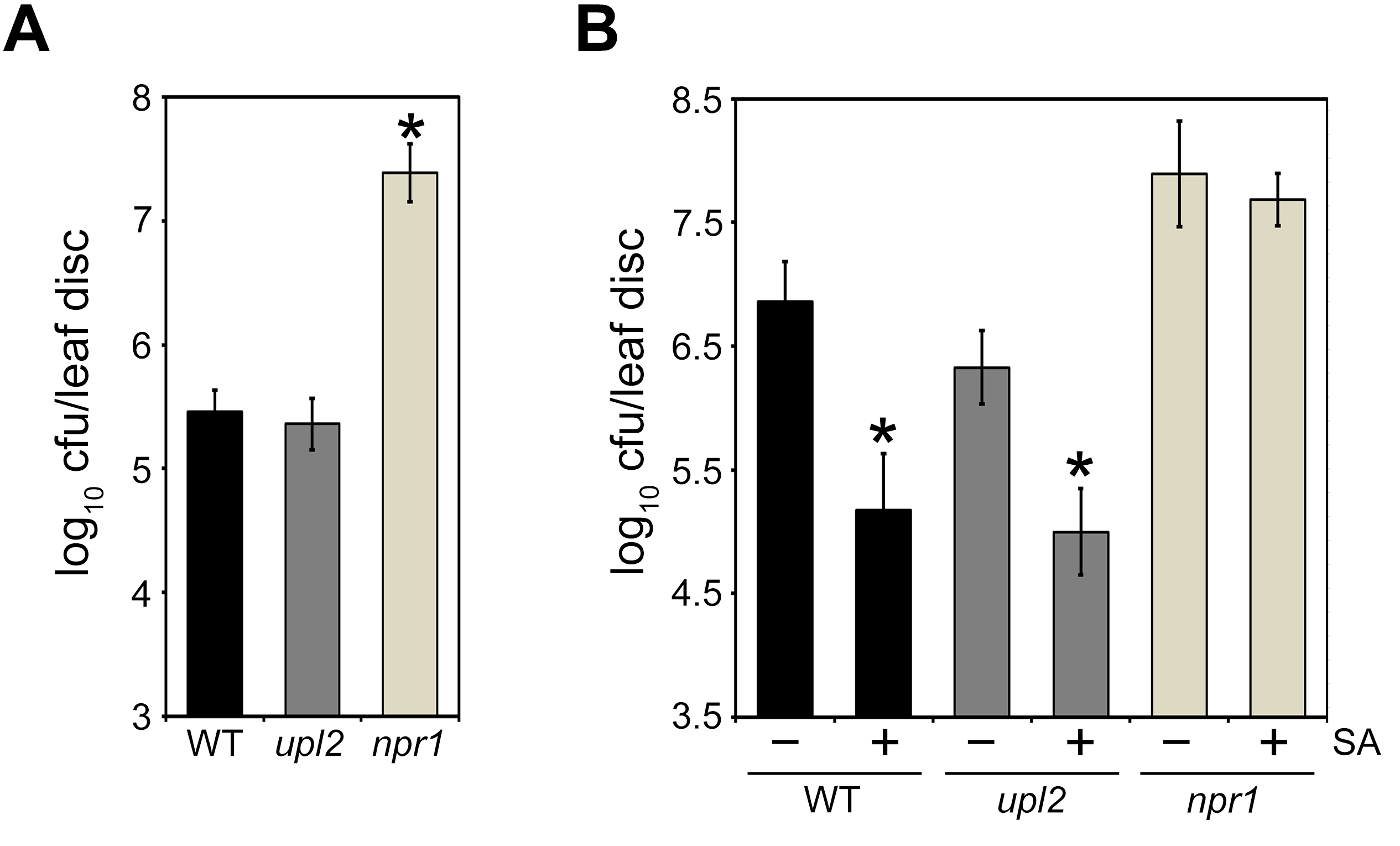

Supplement: S2 Fig — (A) Wild type (WT), upl2 and npr1 plants were infected with Psm ES4326 (5 x 10^5^ cells) and pathogen growth assessed after 4 days. Cfu, colony forming units. Error bars represent statistical 95% confidence limits (n = 8) and asterisks indicate statistically significant differences compared to WT (Tukey-Kramer ANOVA test; α = 0.05, n = 8). (B) Wild type (WT), upl2 and npr1 plants were treated with 0.5 mM SA for 24 hours after which plants were infected with Psm ES4326 (5 x 10^6^ cells) and pathogen growth assessed after 4 days. Cfu, colony forming units. Error bars represent statistical 95% confidence limits (n = 8) and asterisks indicate statistically significant differences between mock (-) and SA (+) treatments for each genotype (Tukey-Kramer ANOVA test; α = 0.05, n = 8). (TIF) [file ppat.1007447.s002.tif]

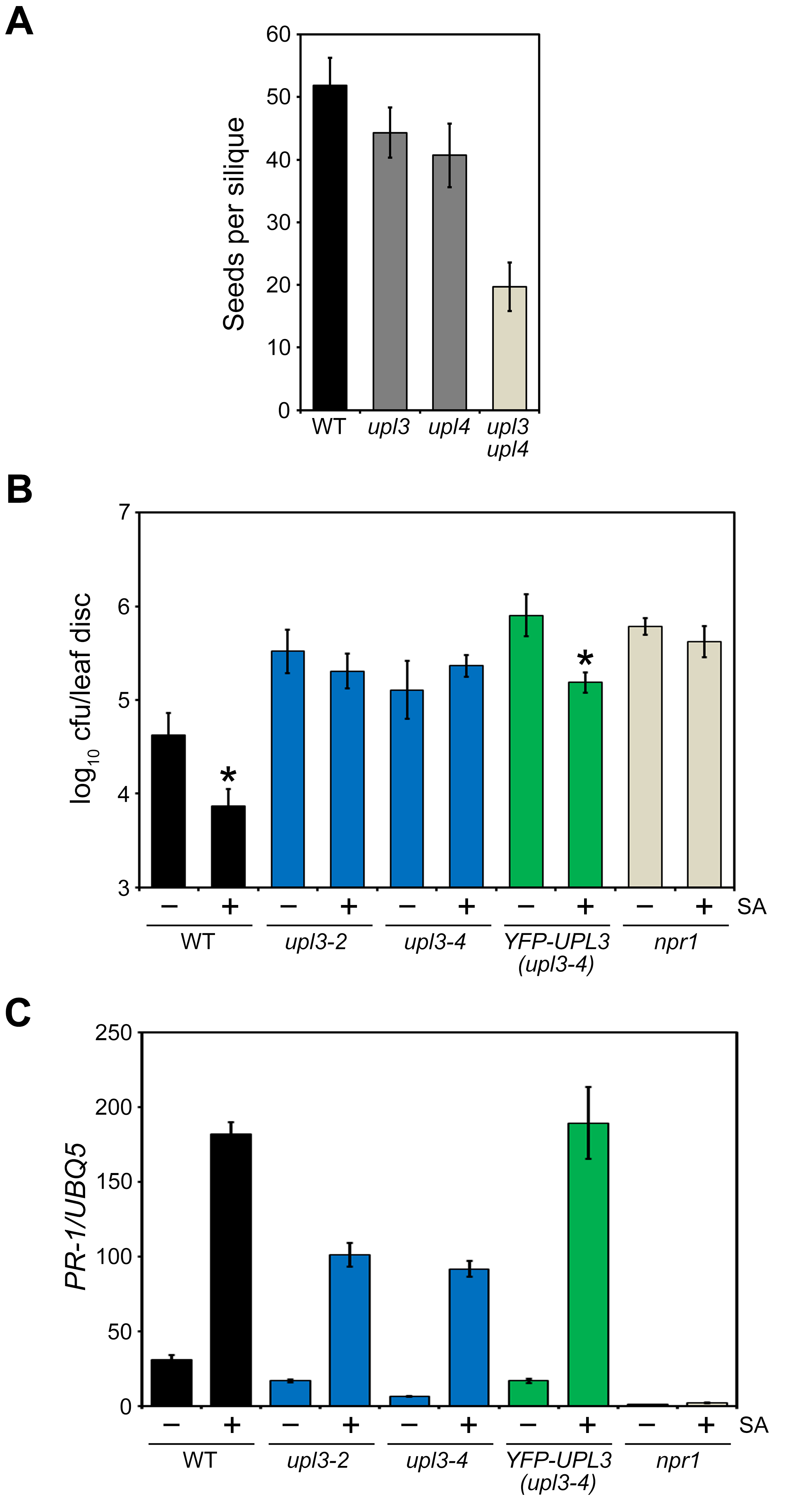

Supplement: S3 Fig — (A) Seeds per silique in adult WT, upl3 and upl4 single, and upl3 upl4 double mutants. Error bars represent SD (n = 10). (B) Wild-type (WT), upl3-2, upl3-4, 35S::YFP-UPL3 (in upl3-4) and npr1 plants were treated with 0.5 mM SA for 24 hours after which plants were infected with Psm ES4326 (5 x 10^6^ cells) and disease symptoms (top panel) as well as pathogen growth (bottom panel) assessed after 3 days. Cfu, colony forming units. Error bars represent statistical 95% confidence limits (n = 8) and asterisks indicate statistically significant differences between mock (-) and SA (+) treatments for each genotype (Tukey-Kramer ANOVA test; α = 0.05, n = 8). (C) Adult wild-type (WT), upl3-2, upl3-4, 35S::YFP-UPL3 (in upl3-4) and npr1 plants were treated with (+) or without (-) 0.5 mM SA for 24 hours. Expression of the immune marker genes PR-1 was analysed by qPCR and normalised against constitutively expressed UBQ5. Error bars represent SD (n = 3). (TIF) [file ppat.1007447.s003.tif]

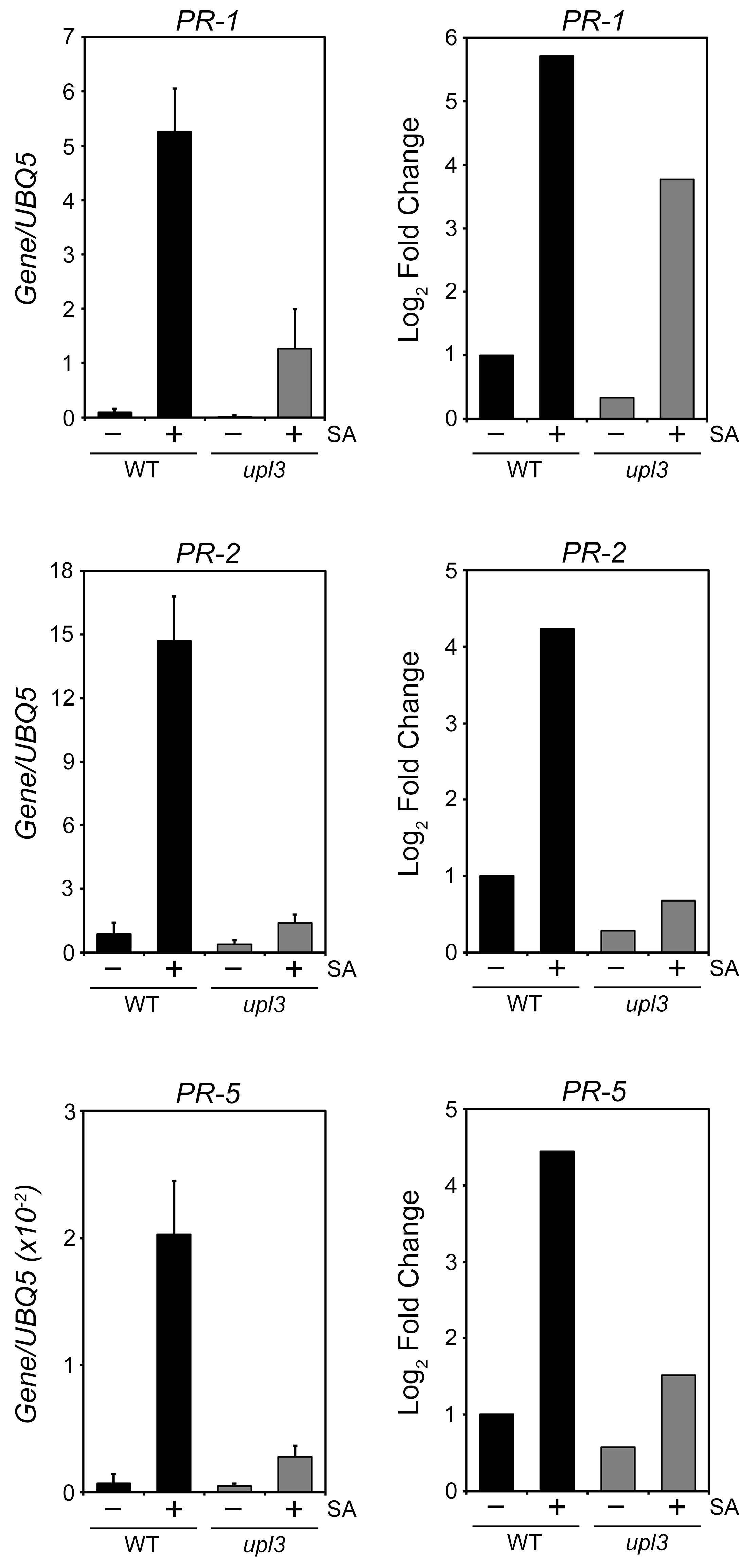

Supplement: S4 Fig — PR gene expression in adult WT and upl3 plants treated for 24 hours with or without 0.5 mM SA was analysed by qPCR (left panels) and by RNA Seq (right panels). Error bars represent SD (n = 3). (TIF) [file ppat.1007447.s004.tif]

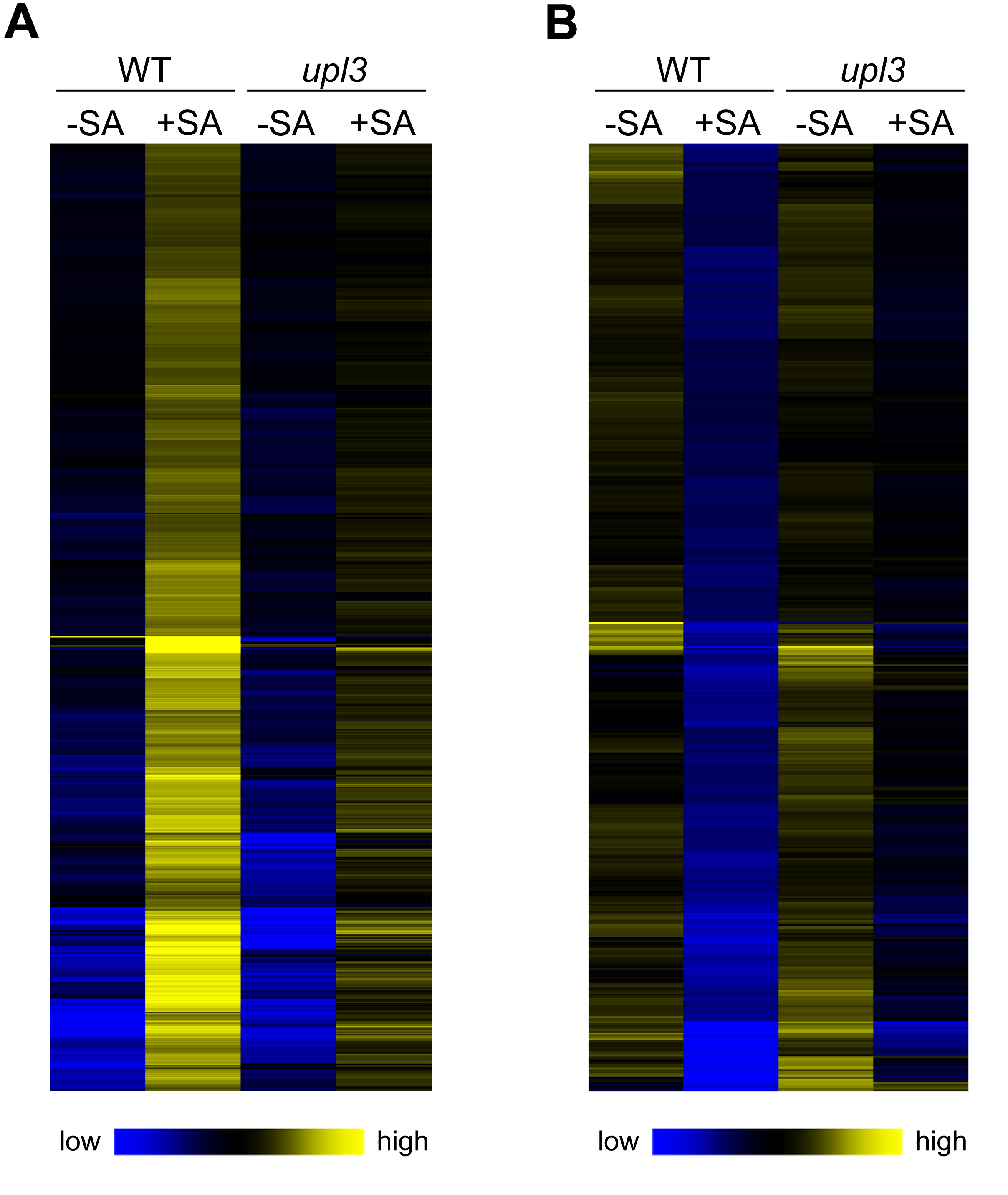

Supplement: S5 Fig — (A) Heat map of 860 SA-induced genes from Venn diagram in Fig 5C for wild-type (WT) and mutant upl3 plants. Highly expressed genes are shown in yellow whereas lowly expressed genes are shown in blue. (B) Heat map of 515 SA-repressed genes from Venn diagram in Fig 5C for wild-type (WT) and mutant upl3 plants. Highly expressed genes are shown in yellow whereas lowly expressed genes are shown in blue. (TIF) [file ppat.1007447.s005.tif]

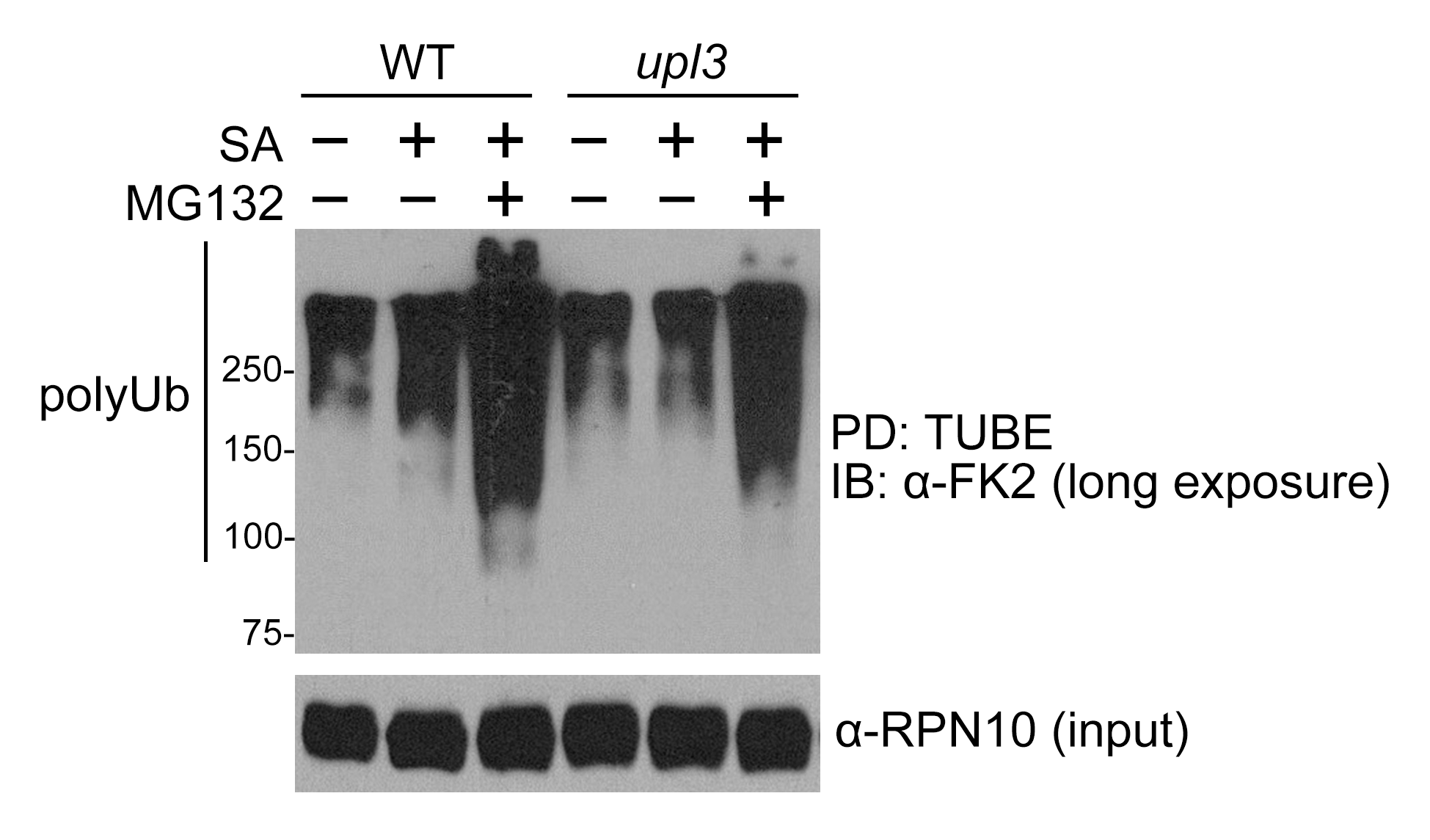

Supplement: S6 Fig — Long exposure of anti-ubiquitin blot shown in Fig 6D. Wild-type (WT) and upl3 plants were treated with (+) or without (-) 0.5 mM SA and 100 μM MG132 for 6 hours. Ubiquitinated proteins were pulled down (PD) using GST-tagged tandem ubiquitin binding entities (TUBE). Total (input) and pulled down proteins were analysed by immunoblotting (IB) using antibodies against ubiquitin conjugates (polyUb, α-FK2) and RPN10. (TIF) [file ppat.1007447.s006.tif]
